# Selective mural cell recruitment of pericytes to networks of assembling endothelial cell-lined tubes

**DOI:** 10.3389/fcell.2024.1389607

**Published:** 2024-06-19

**Authors:** Ksenia Yrigoin, George E. Davis

**Affiliations:** Department of Molecular Pharmacology and Physiology, Morsani College of Medicine, University of South Florida School of Medicine, Tampa, FL, United States

**Keywords:** endothelial cells, capillaries, pericytes, vascular smooth muscle cells, basement membrane matrix deposition, mural cells

## Abstract

Mural cells are critically important for the development, maturation, and maintenance of the blood vasculature. Pericytes are predominantly observed in capillaries and venules, while vascular smooth muscle cells (VSMCs) are found in arterioles, arteries, and veins. In this study, we have investigated functional differences between human pericytes and human coronary artery smooth muscle cells (CASMCs) as a model VSMC type. We compared the ability of these two mural cells to invade three-dimensional (3D) collagen matrices, recruit to developing human endothelial cell (EC)-lined tubes in 3D matrices and induce vascular basement membrane matrix assembly around these tubes. Here, we show that pericytes selectively invade, recruit, and induce basement membrane deposition on EC tubes under defined conditions, while CASMCs fail to respond equivalently. Pericytes dramatically invade 3D collagen matrices in response to the EC-derived factors, platelet-derived growth factor (PDGF)-BB, PDGF-DD, and endothelin-1, while minimal invasion occurs with CASMCs. Furthermore, pericytes recruit to EC tube networks, and induce basement membrane deposition around assembling EC tubes (narrow and elongated tubes) when these cells are co-cultured. In contrast, CASMCs are markedly less able to perform these functions showing minimal recruitment, little to no basement membrane deposition, with wider and shorter tubes. Our new findings suggest that pericytes demonstrate much greater functional ability to invade 3D matrix environments, recruit to EC-lined tubes and induce vascular basement membrane matrix deposition in response to and in conjunction with ECs.

## Introduction

Blood vessels are composed of endothelial cells (ECs) that organize into tubes of varying diameters and supporting mural cells, which include pericytes and vascular smooth muscle cells (VSMCs) (Adams and Alitalo, 2007; Armulik et al., 2011; Stratman and Davis, 2012; Augustin and Koh, 2017; Holm et al., 2018; Majesky, 2018; Kemp et al., 2022). Together ECs and mural cells produce and deposit the vascular extracellular matrix (ECM) that includes basement membrane matrices in capillaries and, also larger vessels, but which in addition have concentric rings of elastic lamellae and interstitial matrices in between layers of VSMCs in larger arteries and veins (Davis and Senger, 2005; Stratman et al., 2009; Wagenseil and Mecham, 2009; Senger and Davis, 2011; Stratman and Davis, 2012; Stratman et al., 2017; Mecham and Ramirez, 2018; Davis and Kemp, 2023; Lin and Davis, 2023). In larger vessels, the adventitial layer includes fibroblasts which also deposit interstitial collagen-rich matrices (Wagenseil and Mecham, 2009; Mecham and Ramirez, 2018; Majesky and Weiser-Evans, 2022). Pericytes are most numerous in the smallest blood vessels including capillaries and post-capillary venules. By far the most abundant vascular tube structures are capillary networks and pericytes are critically required along with ECs to deposit capillary basement membrane matrices (Stratman et al., 2009; Smith et al., 2013; Kemp et al., 2020). Disruption of pericyte recruitment leads to blockade of basement membrane deposition around EC tubes causing them to be wider, shorter, and less stable which makes them more susceptible to pro-regressive stimuli (Kemp et al., 2020). Pericytes and the capillary basement membrane have been strongly implicated in capillary assembly and stabilization, tissue homeostasis, and the control of vascular permeability in sites such as the blood brain barrier (Hellstrom et al., 2001; Nystrom et al., 2006; Armulik et al., 2011). Pericytes are known to be actively recruited to EC tubes through the release of EC-derived paracrine signals which include PDGF-BB, PDGF-DD, endothelin-1 (ET-1), TGFβ1, and HB-EGF (Hirschi et al., 1998; Hirschi et al., 1999; Stratman et al., 2010; Armulik et al., 2011; Kemp et al., 2020; Smart, 2020). These paracrine signals regulate pericyte invasion, recruitment to tubes, proliferation, and survival during vessel morphogenesis. Combined blockade of these EC-derived molecules results in marked diminishment of pericyte recruitment, pericyte proliferation, and basement membrane matrix deposition during capillary assembly (Kemp et al., 2020).

In contrast, there is much less information regarding how VSMCs recruit to EC tubes during developmental events. Both PDGF-BB and HB-EGF have been implicated in this process, which overlap with factors driving pericyte recruitment (Iivanainen et al., 2003; Stratman et al., 2010; Kemp et al., 2020). Much more work has focused on the differentiated status of VSMCs as it relates to their role in contractile functions to control blood pressure, vessel reactivity to vasoconstrictors and vasodilators and their importance in assembly of elastin- and fibrillin-rich vascular extracellular matrices, which represent key properties of larger vessels including arterioles, arteries, and veins (Majesky et al., 2011; Riley and Smart, 2011; Mazurek et al., 2017; Majesky, 2018; Mecham and Ramirez, 2018). Lineage tracing studies have revealed multiple developmental origins of VSMCs from neural crest, mesodermal, and epicardial precursors (Owens et al., 2004; Wang and Olson, 2004; Majesky, 2007; Dong et al., 2008; Wasteson et al., 2008; Majesky et al., 2011; Riley and Smart, 2011; Harmon and Nakano, 2013; Wu et al., 2013; Volz et al., 2015; Sawada et al., 2017; Lupu et al., 2020). In addition, other work suggests that pericytes appear to be a precursor of VSMCs in both the coronary and renal vasculatures (Volz et al., 2015). Genetic analyses of pericytes and VSMCs reveal considerable overlaps in the mRNA expression profiles of these two types of mural cells (He et al., 2016; He et al., 2018; Bordenave et al., 2020; Muhl et al., 2020; Orlich et al., 2022).

In the current study, we have performed functional assays to compare the ability of two mural cell types, pericytes and CASMCs (as a representative of VSMCs) to co-assemble with ECs during tube network formation in 3D collagen matrices. In addition, we have assessed the ability of these mural cells to respond to EC-derived factors and invade 3D collagen matrices; a necessary step for mural cells to recruit to developing EC tube networks. Our findings reveal a major functional distinction between pericytes vs. CASMCs in that pericytes selectively demonstrate the ability to markedly invade 3D collagen matrices, strongly recruit to forming EC tube networks, and co-regulate the assembly of vascular basement membranes along the abluminal surface of EC tubes. In contrast, CASMCs showed minimal responsiveness to EC-derived factors in invasion assays or during EC tube assembly, where poor recruitment occurred with low levels of basement membrane matrix deposition. Basement membrane matrix deposition robustly occurs along the abluminal surface of elongated and narrow EC tubes during EC-pericyte tube co-assembly but is much less apparent in EC-CASMC co-cultures, where the EC tubes are significantly less elongated and show greater EC tube widths. Overall, our detailed functional analyses suggest that pericytes demonstrate much greater abilities to invade ECM matrices, recruit to developing EC tube networks, and induce vascular basement membrane matrix assembly in conjunction with ECs to facilitate EC tube maturation and stabilization events.

## Methods

The authors declare that all supporting data are available within the article (and its Supplementary Material). Materials used in this study are listed below.

### Cell culture

Human umbilical vein ECs were used from passage 3 to 6. Human aortic ECs were used from passage 3 to 6. Human ECs and coronary artery-derived smooth muscle cells (CASMCs) were obtained from Lonza (Basel, Switzerland), while human brain vascular pericytes (HBVP) were obtained from ScienCell (Carlsbad, CA). HBVPs were used from passage 4 to 12 and were labeled with green fluorescent protein (GFP). CASMCs were used from passage 3 to 6 and were GFP-labeled. All cells were passaged on gelatin-coated flasks and grown in our own Supermedia, with M199 as a base, 20% fetal bovine serum, bovine hypothalamus extract, heparin sodium salt, gentamicin, and amphotericin B and prepared as described (Koh et al., 2008). Cells were grown in incubators set at 37°C and 5% CO_2_.

### Nucleic acid extraction and qPCR analysis

After reaching confluency, HBVP and CASMC cultures were lysed using TRIzol (Zymo Research, Irvine, CA, #R2051) to isolate total RNA. cDNA was produced using the Protoscript–First strand cDNA synthesis kit (# E6300s) from New England Biolabs (Ipswich, MA). qPCR was done using TaqMan Fast Advanced Master Mix (2x) and endogenous control *gapdh* TaqMan Assay (Hs02786624_g1) from ThermoFisher Scientific (Waltham, MA). Multiplex analysis was performed to compare levels of target genes to endogenous control. Target gene TaqMan reagents used are as follows: *cspg4* (Hs00361541_g1), *pdgfrb* (Hs01019589_m1), *ednra* (Hs03988672_m1), *tgfbr1* (Hs00610320_m1), *timp3* (Hs00165949_m1), *tbx3* (Hs00195612_m1), *eln* (Hs00355783_m1), *lox* (Hs00942483_m1), *adamts4* (Hs00192708_m1), *tgm2* (Hs01096681_m1), *prg4* (Hs00981633_m1), *notch3* (Hs01128537_m1), *tagln* (Hs01038777_g1), *cald1* (Hs00921987_m1), *cnn1* (Hs00959434_m1), *smtn* (Hs01022255_g1), *acta2* (Hs00426835_g1). Reactions were mixed with corresponding cDNA samples, placed in 96-well 0.1 mL plates and analyzed using an Applied Biosystems Quant Studio 3 real-time PCR machine (ThermoFisher Scientific).

### Invasion assays

Type I collagen matrices (2.5 mg/mL) were polymerized and equilibrated at 37°C in 5% CO_2_ for 45 min. After polymerization, mural cells (at 1.4 × 10^6^ cells/mL) were seeded on top of gel with reduced-serum II supplement (RSII) in M199 (RSII media). Molecules for different conditions were added directly on top of the polymerized gel and resuspended cells. Mural cells were then cultured over a period of 72 h and waited for their invasion response. After 72 h, pericyte or VSMCs cultures were fixed in 3% glutaraldehyde and stained with 0.1% toluidine blue in 30% methanol for nonfluorescent imaging or in 3% paraformaldehyde to be immunostained for fluorescent imaging. Pericyte invasion was quantified and imaged at predetermined depths. Four images per depth at 2 to 3 depths (8–12 fields per experiment); ≥3 validating experimental replicates were conducted in total.

### Vessel network assembly assay

Human umbilical vein ECs or human aortic ECs (at 2 × 10^6^ cells/mL) and pericytes or VSMCs (0.4 × 10^6^ cells/mL) were cocultured in 2.5 mg/mL type I collagen matrices in 96-well half area plates. After polymerization, cocultures were fed with 1x M199 media containing RSII (containing insulin), FGF (fibroblast growth factor)-2 at 50 ng/mL, SCF (stem cell factor) at 40 ng/mL, IL-(interleukin)-3 at 40 ng/mL, and SDF (stromal cell-derived factor)-1α at 40 ng/mL as described (Stratman et al., 2011; Smith et al., 2013; Bowers et al., 2016). FGF-2 was obtained from Gibco (Grand Island, NY), while SCF, IL-3, and SDF-1α were obtained from R&D Systems (Minneapolis, MN). These cultures assembled for a period of 0–72 h or 0–120 h and then fixed in 3% paraformaldehyde. ECs were primed with VEGF (R&D Systems) at 40 ng/mL for 16 h (Bowers et al., 2020), prior to suspending them in 3D collagen matrices in the presence of pericytes or CASMCs. To quantify mural cell recruitment, 5 pictures were taken from predetermined locations per experiment fixed at 72 h, and ≥3 validating experimental replicates were conducted in total for all quantitative data. Pericyte recruitment was assessed by 2 criteria: (1) To be recruited, most of the mural cell body must be on an EC tube and (2) the mural cell must be elongated on that tube (rounded up cells did not qualify as recruited). To quantify vessel width and length 120-h cocultures were fixed with 3% glutaraldehyde and stained with 0.1% toluidine blue in 30% methanol for nonfluorescent imaging. Four images per well and ≥3 wells per condition were taken. Widths and lengths were consequently measured and analyzed.

### Immunostaining of 3D cultures

After fixation in 3% paraformaldehyde, collagen gels were washed in a Tris-glycine buffer solution for 1 h. In some cases, a 1% Triton-X100 solution was added for 1 h. To analyze basement membrane deposition, we did not permeabilize the fixed cultures. Gels were then put into a blocking solution containing 5% serum specific to the secondary antibody (either goat or rabbit for this work) for 1 h. Later, the primary antibody was added directly into blocking solution and allowed to incubate overnight. After incubation, the solution containing the primary antibody was removed, and the gels were washed several times with Tris-buffered saline. Blocking solution containing 5% serum was added with secondary fluorescent antibody. After 2 h, this solution was removed and washed again several times with Tris-buffered saline. Samples could then be imaged by immunofluorescence microscopy. For immunostaining, we utilized the following basement membrane matrix component antibodies: Fibronectin 1:200 (Rockland, Philadelphia, PA, #6004011170.5), Laminin 1:200 (Sigma, St. Louis, MO, #071M4867), Perlecan 1:200 (Invitrogen, Carlsbad, CA, #134400), Collagen IV derived from M3F7 Hybridoma 1:4 (Developmental Studies Hybridoma Bank, Iowa City, IA), Nidogen-1 1:200 (R&D Systems, Minneapolis, MN, #AF2570), Nidogen-2 1:200 (R&D Systems, #AF3385). To image ECs, we immunostained cultures with anti-CD31 1:200 (Dako, Glostrup, Norway, #M0823).

### Microscopy and imaging

Images for quantification of pericyte recruitment and pericyte invasion were obtained using DIC images of ECs, which were then overlaid with fluorescent GFP (green fluorescent protein) mural cells on an Olympus CKX41 microscope (Olympus, Tokyo, Japan) and imaging software, DP Controller/DP Manager version 3.2.1.276 (Olympus). Time-lapse movies on living cells, as well as immunofluorescence microscopy, were both obtained using a DMI6000B microscope with environmental chamber (Leica Microsystems, Wetzlar, Germany) and controlled using MetaMorph 7.8 software (Molecular Devices, San Jose, CA). The magnification of ×10 objective was used for all movies. To create timelapse movies, images were taken every 10 min with a monochromatic Hamamatsu ORCA-ER C4742-80 camera (Hamamatsu City, Japan) in different stage locations over a period of 0–72 h. These images could then be compiled into a movie using MetaMorph software. MetaMorph was also used to evaluate EC tube widths and EC tube lengths. Movies were stabilized and edited using Adobe Creative Cloud: Adobe After Effects (Adobe Systems, San Jose, CA). Confocal images were obtained using a Leica SP8 LIGHTNING White light laser confocal scanning microscope. All images were obtained using a ×10 HC PL APO, 0.4NA, WD 2.2 mm lens. Confocal reconstructions and imaging were created using either LAS X (Leica Microsystems) or Fiji (ImageJ). The ImageJ version is 1.45f (https://imagej.nih.gov/ij/index.html, last accessed 1 July 2023).

### Statistics

Student *t* tests were performed using Prism 10 (GraphPad Software, Boston, MA) to assess statistical significance between means of various conditions. Data were analyzed for normality and equal variances were obtained. When it was necessary to cross compare the means of multiple conditions within a given experiment, GraphPad (Prism 10) was used for ANOVA with follow up *post hoc* Tukey tests. Alpha value = 0.05 was set as the minimum statistical significance. All experiments were performed with ≥3 validating experimental replicates in total.

## Results

### Comparative analysis of human mural cell recruitment to developing networks of human EC-lined tubes under defined media conditions

**FIGURE 1:**
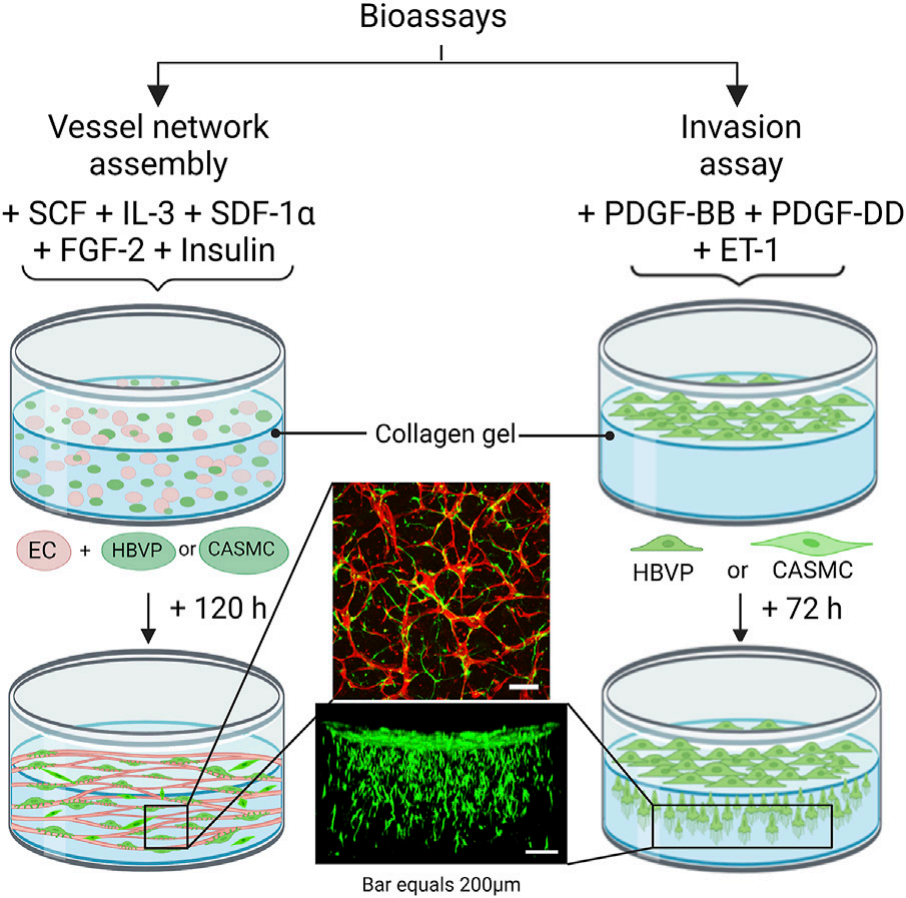
Schematic diagram illustrating bioassays which are utilized to evaluate human mural cell functions during assembly of blood vascular tube networks. These assay models are performed using serum-free defined media and using three-dimensional (3D) collagen matrices in 96 well half-area microplates. The vascular network assembly model consists of seeding the cells within 3D collagen gels and a combination of five growth factors is added to the culture media: stem cell factor (SCF), interleukin-3 (IL-3), stromal-derived factor-1-alpha (SDF-1α), fibroblast growth factor-2 (FGF-2) and insulin. Endothelial cells (ECs), human brain vascular pericytes (HBVP) or coronary artery smooth muscle cells (CASMCs) were added in these co-cultures, and they were later fixed and immunostained after 120 h. Both the pericytes and CASMCs are labeled with green fluorescent protein (GFP) and ECs are visualized by immunostaining with anti-CD31 (red) antibodies. The invasion assay consists of seeding the mural cells on top of the gel along with the EC-derived factor treatment which includes platelet-derived growth factor (PDGF)-BB, PDGF-DD, and endothelin-1 (ET-1). Cultures are later fixed after 72 h and imaged from the side using confocal microscopy.

In this work, we have addressed an important and unresolved question which evaluates the functional ability of the two key human mural cell types, pericytes and VSMCs, to recruit to assembling EC-lined tube networks and induce stabilization of these tubes through basement membrane matrix deposition. For this analysis, we have utilized serum-free defined models in 3D collagen matrices developed by our laboratory to investigate EC tubulogenesis, EC sprouting behavior, mural cell invasive ability, and EC-mural cell tube co-assembly (Stratman et al., 2011; Bowers et al., 2016; Kemp et al., 2022) (Figure 1). We evaluated human brain vascular pericytes (HBVP) vs. coronary artery smooth muscle cells (CASMC) (as a model for VSMCs) for our analysis, and the cell types were grown up using the same culture conditions. Pericytes and CASMCs are known to be associated with either the capillary or coronary arterial vasculature, respectively, but it is not clear whether these differentiated mural cells can directly recruit to vascular tubes during vascular assembly. A lineage tracing study suggested that pericytes may represent the developmental precursors for CASMC as well as VSMCs in the kidney (Volz et al., 2015).

### Human pericytes and CASMCs show similarities in gene expression of mural cell markers but also demonstrate key differences

**FIGURE 2:**
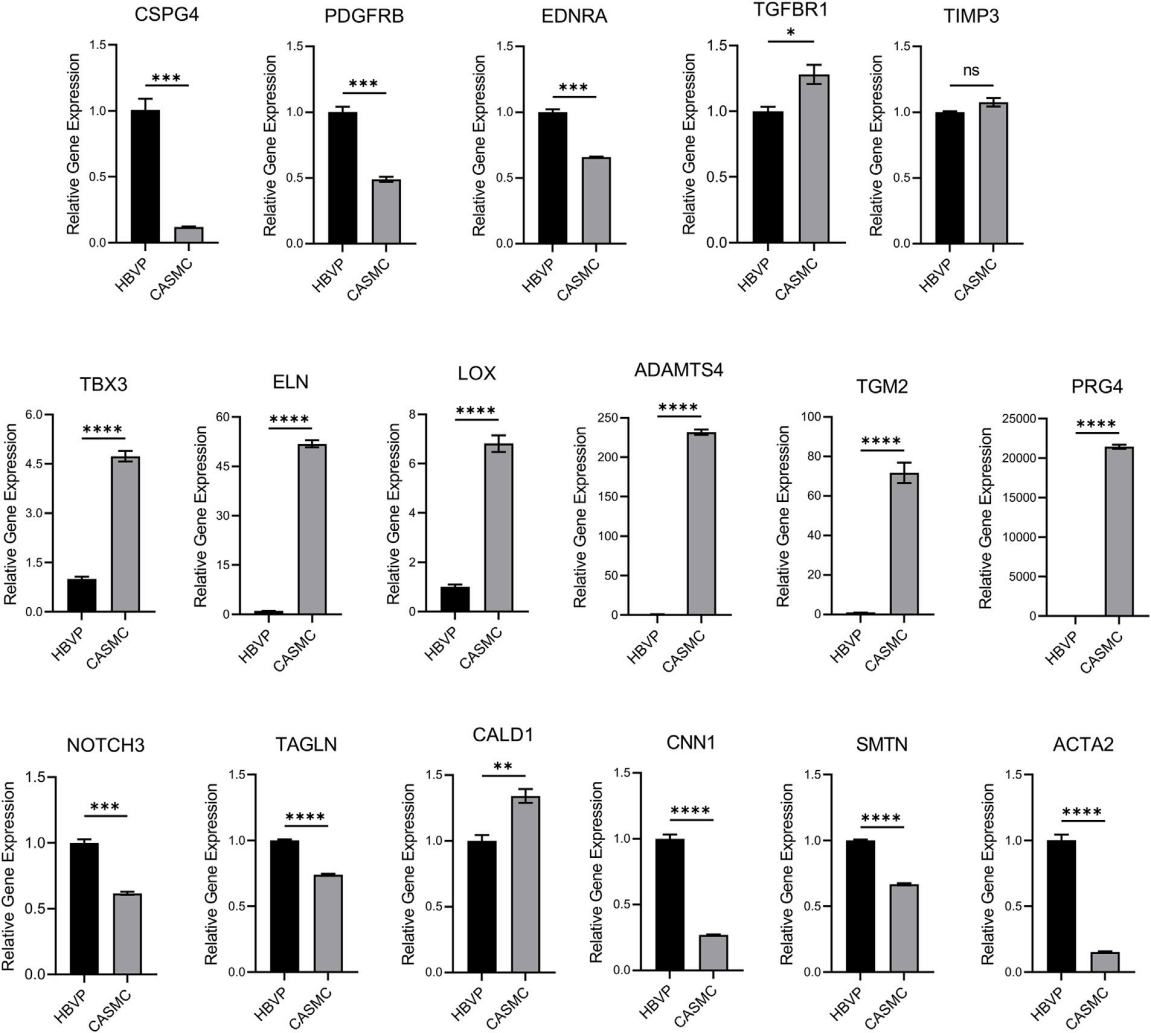
Comparative mRNA expression in pericytes vs. coronary artery smooth muscle cells of selected mural cell genes. Quantitative PCR detection of the relative levels of various mural cell genes comparing human brain vascular pericytes (HBVPs) and coronary artery smooth muscle cells (CASMCs). The following genes were evaluated: Chondroitin Sulfate Proteoglycan 4 (CSPG4), Platelet Derived Growth Factor Receptor B (PDGFRB), Endothelin 1 Receptor A (EDNRA), Transforming Growth Factor Beta Receptor 1 (TGFBR1), TIMP Metallopeptidase Inhibitor 3 (TIMP3), Neurogenic locus notch homolog protein 3 (NOTCH3), Elastin (ELN), Transgelin (TAGLN), Caldesmon 1 (CALD1), Calponin 1 (CNN1), Smoothelin (SMTN), T-Box Transcription Factor 3 (TBX3), Smooth Muscle Actin 2 (ACTA2), Lysyl Oxidase (LOX), ADAM Metallopeptidase with Thrombospondin Type 1 Motif 4 (ADAMTS4), Transglutaminase 2 (TGM2), and Proteoglycan 4 (PRG4). (*n* = 3), * indicates significance at *p* ≤ 0.05, ** indicates significance at *p* < 0.01, *** indicates significance at *p* < 0.001, and **** indicates significance at *p* < 0.0001.

Because of the many similarities that exist between pericytes and VSMCs, there would be an expectation that they may be functionally capable of responding, like pericytes, to stimuli released by ECs to promote EC-mural cell tube co-assembly. As an initial approach, we selected genes that show similarities but also reveal differences via qPCR analyses, with *gapdh* as an internal control (Figure 2). We screened mural cell marker genes including Chondroitin Sulfate Proteoglycan 4 (*cspg4*), Platelet Derived Growth Factor Receptor B (*pdgfrb*), Endothelin 1 Receptor A (*ednra*), Transforming Growth Factor Beta Receptor 1 (*tgfbr1*) and Tissue inhibitor of metalloproteinases-3 (*timp3*). We also evaluated smooth muscle marker genes including Elastin (*eln*), Notch3 (*notch3*), Transgelin (*tagln*), Caldesmon 1 (*cald1*), Calponin 1 (*cnn1*), Smoothelin (*smtn*), and Smooth Muscle Actin 2 (*acta2*). We observed that *cspg4*, *pdgfrb*, *ednra*, *notch3*, *tagln*, *cnn1*, *smtn*, and *acta2* show significantly higher expression in pericytes compared to CASMCs, while *tgfbr1* is expressed at higher levels in CASMCs vs. pericytes, while *timp3* shows equivalent expression (Figure 2). As expected, *eln* was expressed at much higher levels in CASMCs with little to no expression in pericytes (Figure 2). *cald1* was also expressed at higher levels in CASMCs than in pericytes (Figure 2). We also screened other genes that we suspected might show higher expression in VSMCs, including the transcription factor, tbx3 (De Bono et al., 2023), and the vascular ECM-related genes, *eln*, Lysyl Oxidase (*lox*), ADAM Metallopeptidase with Thrombospondin Type 1 Motif 4 (*adamts4*), Transglutaminase 2 (*tgm2*), and Proteoglycan 4 (*prg4*) that play a role in arterial vascular wall ECM assembly and stabilization (Wagenseil and Mecham, 2009; Mecham and Ramirez, 2018; Lin and Davis, 2023); and this was indeed the case (Figure 2). Our results suggest that even though pericytes and VSMCs are mural cells that may originate from a similar or related progenitor cell, they differ in their genetic expression of key regulatory proteins and mural cell markers, indicating that they could demonstrate functional differences in the ability to recruit to EC-lined tubes during blood vessel assembly.

### Pericytes respond to endothelial-derived factors and dramatically invade into 3D collagen matrices while CASMCs demonstrate markedly less invasiveness

**FIGURE 3:**
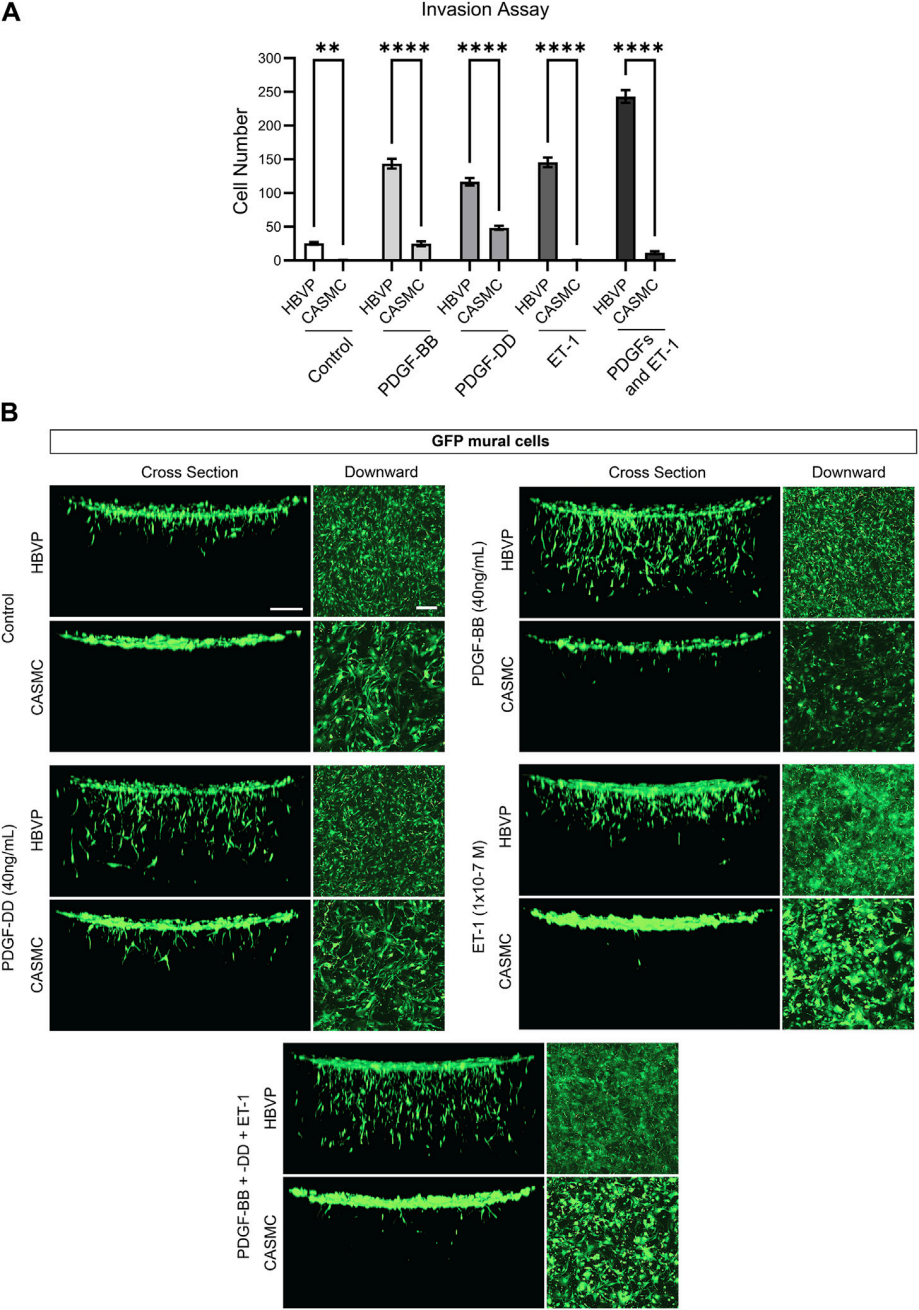
Marked ability of pericytes to invade 3D collagen matrices in response to endothelial-derived factors compared to coronary artery smooth muscle cells, which show strongly diminished invasive ability. **(A)** Green fluorescent protein (GFP)-labeled human brain vascular pericytes (HBVP) or coronary artery smooth muscle cells CASMCs were left untreated or were treated with the indicated endothelial derived factors PDGF (platelet-derived growth factor)-BB, PDGF-DD, ET-1 (endothelin 1) individually or these factors in combination and seeded on the surface of 3D collagen matrices. After 72 h, the invasion response was quantitated (*n* = 5). * indicates significance relative to control HBVP, *p* ≤ 0.05; **** indicates significance of *p* ≤ 0.0001. **(B)** Fixed cultures were imaged using confocal z-stack, 3-dimensional (3D) reconstructions of the indicated GFP-labeled mural cells. These were viewed from the top as a downward view or rotated 90° and observed from the side as a cross-sectional view. Bar equals 200 μm.

Previously, we reported that pericytes markedly respond to the EC-derived factors (i.e., PDGF-BB, PDGF-DD, and ET-1, and their combination) to invade into 3D collagen gels (Kemp et al., 2020). We identified these factors through our serum-free invasion assays and, also revealed their critical functional roles in stimulating pericyte recruitment to developing EC tube networks, by blocking the ligands or their receptors (Kemp et al., 2020). Here, we have compared the invasion response of the two mural cells side by side to the EC-derived factors which drive EC tube recruitment. To accomplish this, we added PDGF-BB, PDGF-DD, ET-1, or all three in combination to determine how CASMCs invade into 3D collagen matrices compared to pericytes (Figure 3). Pericytes strongly invade in response to PDGF-BB, PDGF-DD, ET-1 and their combination, while CASMCs show markedly less invasion (Figure 3). Additionally, the invasion assay was also evaluated by confocal z-stack reconstructions of GFP-pericytes or GFP-VSMCs migrating downward into 3D collagen gels (Figure 3). We rotated the image 90° for a side-view cross section of the invasion response. Interestingly, PDGF-BB and PDGF-DD were able to elicit a weak invasive response from the CASMCs when compared to its untreated control, while ET-1 did not induce invasion. Pericytes invaded to a much greater degree in response to the individual factors and, also when treated with the combination of PDGF-BB, PDGF-DD, and ET-1. However, this latter response was not observed using CASMCs (Figure 3). This data suggests that EC-derived factors that are known to be critical and essential for pericyte invasion, recruitment, and proliferation during capillary assembly (Kemp et al., 2020), do not elicit the same response from CASMCs.

### Pericytes markedly recruit to assembling EC-lined tubes and induce basement membrane deposition while CASMCs demonstrate minimal recruitment with reduced basement membrane matrix deposition

**FIGURE 4:**
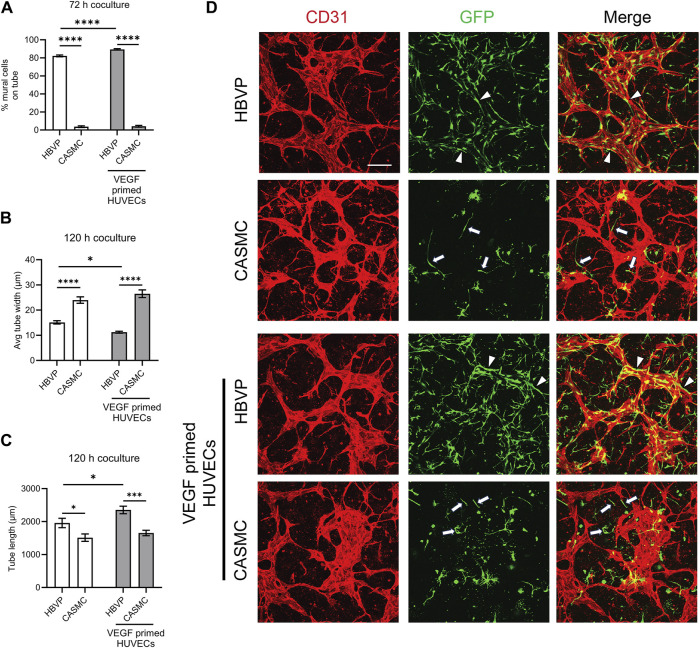
Pericytes recruit to developing EC-lined tube networks and affect EC tube remodeling to generate narrow and elongated tubes, while coronary artery smooth muscle cells show minimal recruitment creating wider and shortened EC tube networks. Human umbilical vein ECs (HUVECs) were utilized for these experiments and were either left untreated or primed by vascular endothelial cell factor (VEGF). Untreated ECs or ECs primed with VEGF were co-cultured with either GFP-labeled pericytes or coronary artery smooth muscle cells (CASMCs) and then cultured and fixed at 120 h to assess the degree of mural cell recruitment and the impact of this recruitment on EC tube remodeling. **(A)** Percentage of human brain vascular pericytes (HBVP) vs. CASMC recruited to EC tubes (untreated or following VEGF priming of ECs) at 72 h. *n* = 17; **** indicates significance at *p* ≤ 0.0001. **(B)** Average tube width of 120 h EC tube networks (untreated or following VEGF priming of ECs) and pericyte or CASMC cocultures; *n* = 15, * indicates significance at *p* ≤ 0.05; **** indicates significance at *p* ≤ 0.0001. **(C)** EC (untreated or following VEGF priming of ECs) + pericyte or CASMC cocultures were quantitated for total tube length. *n* = 15; * indicates significance at *p* ≤ 0.05; *** indicates significance at *p* ≤ 0.001. **(D)** EC (untreated or following VEGF priming of ECs) + pericyte or CASMC cocultures were established and fixed after 120 h. Cocultures were immunostained with CD31 (red) and mural cells are labeled with green fluorescent protein (GFP) and were imaged by confocal microscopy. Representative overlay images are illustrated in the right panels, while the middle panels show mural cells alone, and the left panels show EC tube networks alone. Arrowheads indicate mural cells that have recruited to EC tubes, while arrows indicate mural cells that have not recruited to the tubes. Bar equals 200 μm.

After we observed that CASMCs do not respond as effectively to known EC-derived factors when compared to the response made by pericytes, we performed experiments to compare both mural cell’s ability to recruit to EC-lined tubes using our 3D bioassays for vessel assembly (Figures 1, 4). After 72 h, co-cultures were fixed and evaluated for the extent of mural cell recruitment. We observed that assembling EC-lined tubes recruit pericytes to a dramatically greater extent than CASMCs (Figure 4A). The lack of CASMC recruitment to the EC tubes led to increased tube widths and decreased tube lengths compared to the pericyte-associated EC-lined tube networks (Figures 4B, C), which occurs with EC tubes over time in the absence of pericytes (Stratman et al., 2009; Kemp et al., 2020). In addition, we performed real-time video analysis of this differential mural cell invasion and recruitment response over a 72 h period (Supplementary Videos S1–S4). Pericyte recruitment to EC tubes is readily observed in these videos (Supplementary Videos S1, S2), while CASMCs show little movement or recruitment toward EC tubes (Supplementary Videos S3, S4).

In a separate experiment, we primed ECs with vascular endothelial growth factor (VEGF) for 16 h, followed by addition of five defined growth factors (SCF, IL-3, SDF-1α, FGF-2, and insulin) during the co-culture assay. Sequential addition of this combination of growth factors is known to enhance EC tubulogenesis, pericyte recruitment, and pericyte-induced capillary basement membrane deposition (Stratman et al., 2011; Bowers et al., 2020). Also, VEGF exposure of ECs is known to stimulate arteriogenesis (Lawson et al., 2002), a process whereby arteries demonstrate selective VSMC association, with few if any pericytes. Thus, one possibility is that VEGF priming could result in enhanced CASMC recruitment compared to pericyte recruitment to forming EC tubes. Instead, we observe that pericyte recruitment is enhanced to VEGF-primed EC tubes, while CASMCs still fail to recruit to these tubes (Figures 4A, D). In addition, we found that EC tubes in cocultures with pericytes have significantly thinner and longer tubes compared to those with the CASMCs due to the diminished recruitment response (Figures 4B–D). Also, priming with VEGF resulted in thinner and longer tubes in the pericyte coculture, but not in the VSMC coculture (Figures 4B, C). We also performed real-time video analysis to visualize the invasive ability and recruitment of GFP-labeled mural cells to VEGF-primed EC-lined tubes (analyzed from 0 to 72 h) (Supplementary Videos S5–S8). The movies demonstrate that pericytes markedly recruit to forming VEGF-primed EC-lined tubes, while CASMCs show little responsiveness or recruitment to these developing tubes.

**FIGURE 5:**
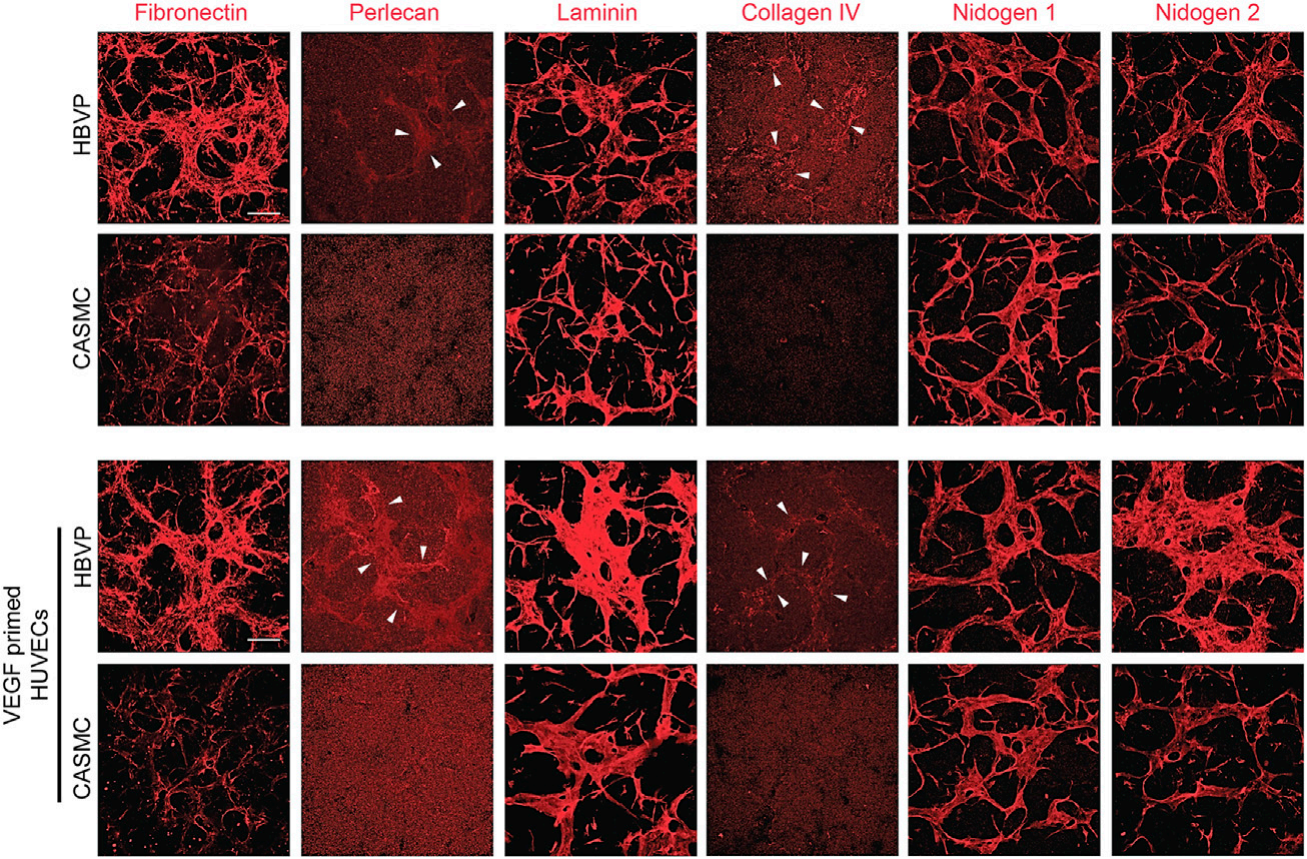
Pericyte recruitment to developing EC tube networks markedly induces basement membrane matrix protein deposition, while, in contrast, coronary artery smooth muscle cells fail to recruit to tubes leading to reduced basement membrane deposition. Human umbilical vein endothelial cells (HUVECs) were utilized for these experiments and were either left untreated or primed with vascular endothelial cell factor (VEGF). Untreated ECs or ECs primed with VEGF were co-cultured with either GFP-labeled pericytes or coronary artery smooth muscle cells (CASMCs) in 3D collagen matrices and then fixed after 120 h. Immunostaining for the indicated basement membrane matrix proteins was performed in the absence of detergent and the stained cultures were imaged by confocal microscopy to assess extracellular deposition of basement membrane components. Arrowheads indicate deposition of perlecan or collagen type IV. Bar equals 200 μm.

Previously, we reported that pericyte recruitment to control or VEGF-primed EC-lined tubes is necessary for capillary basement membrane matrix deposition (Stratman et al., 2011; Bowers et al., 2020). Blockade of pericyte recruitment caused strongly reduced basement membrane formation with concomitant widening and shortening of EC tubes (Bowers et al., 2020; Kemp et al., 2020). Here, we have evaluated the impact of the presence of pericytes vs. CAMSCs in co-cultures with forming EC tube networks (using control or VEGF-primed ECs) on basement membrane assembly. Our results revealed marked basement membrane matrix assembly around control or VEGF-primed EC tubes, when pericytes were present, but not in the presence of CASMCs, due to their inability to recruit to EC tubes in sufficient numbers (Figure 5). Strong deposition of fibronectin, perlecan, laminin, collagen type IV, laminin, nidogen 1 and nidogen 2 was observed in the presence of pericytes, while substantial reductions in fibronectin, perlecan, collagen type IV, and nidogen 2 deposition were observed when CASMCs were present compared to the pericyte co-cultures with either control ECs or VEGF-primed ECs (Figure 5). The differences with nidogen 2 deposition are more striking when the VEGF-primed ECs are used (Figure 5). There appears to be comparable levels of laminin and nidogen 1 deposited using the two mural cell types when cultured with control vs. VEGF-primed ECs. In our previous studies, there is some EC background deposition of laminin and nidogen 1 in the absence of pericytes or blockade of pericyte recruitment (Stratman et al., 2009; Kemp et al., 2020). Thus, the presence of CASMCs does appear to have some ability to increase laminin and nidogen 1 deposition, but otherwise show substantial reductions in the other key basement membrane matrix components. There is a low degree of CASMC recruitment and interaction with the widened EC tubes in this case, which might be the reason for this effect. Overall, this data provides considerable evidence that the two mural cells, pericytes and CASMCs, are functionally distinct in their responsiveness to the EC-derived factors, PDGF-BB, PDGF-DD, and ET-1, recruitment to assembling EC-lined tubes, stabilization of tube widths, elongation of tube lengths, or deposition of basement membrane matrix components.

### Pericytes strongly recruit to developing aortic EC tube networks and induce basement membrane deposition, while CASMCs demonstrate substantially less recruitment and basement membrane assembly during vessel formation

**FIGURE 6:**
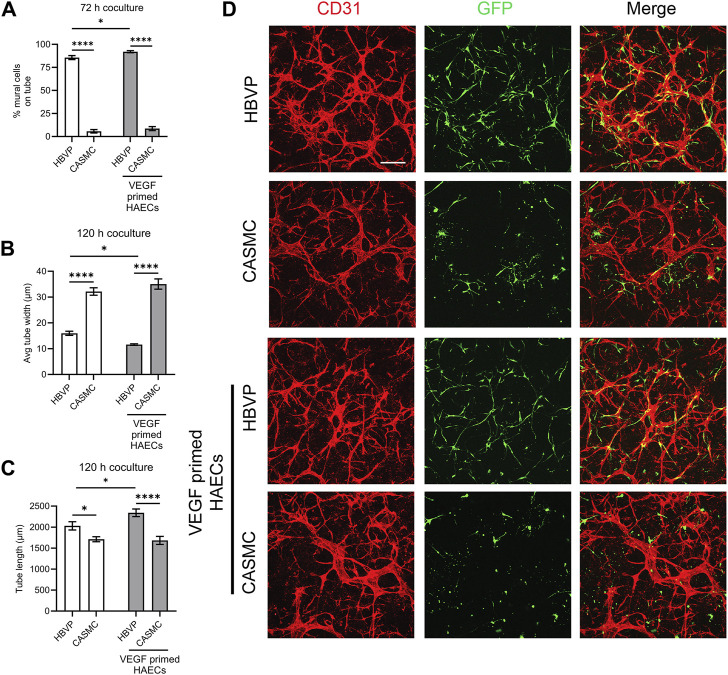
Pericytes markedly recruit to aortic EC-lined tube networks while coronary artery smooth muscle cells show minimal recruitment ability. Human aortic endothelial cells (HAECs) were utilized for these experiments and were either left untreated or were primed with vascular endothelial cell factor (VEGF). Untreated ECs or VEGF-primed ECs were co-cultured with either GFP-labeled pericytes or coronary artery smooth muscle cells (CASMCs) and then fixed after 120 h. **(A)** Percentage of HBVP vs. CASMC recruited to EC tubes (untreated or following VEGF priming of ECs) at 72 h coculture; *n* = 17. * indicates significance at *p* ≤ 0.05; * indicates significance at *p* ≤ 0.0001. **(B)** Average tube width of 120 h HUVEC (untreated or following VEGF priming of ECs) and HBVP or CASMC coculture; *n* = 15. * indicates significance at *p* ≤ 0.05; * indicates significance at *p* ≤ 0.0001. **(C)** HAEC (untreated or following VEGF priming of ECs) + HBVP or CASMC cocultures were quantitated for total tube length; *n* = 15. * indicates significance at *p* ≤ 0.05; * indicates significance at *p* ≤ 0.0001. **(D)** HAEC (untreated or following VEGF priming of ECs)—HBVP or CASMC cocultures were established and fixed after 120 h. Cocultures were immunostained with anti-CD31 antibodies (red) and mural cells are labeled with GFP (green). Overlay images are shown in the right panels. Bar equals 200 μm.

Another possibility that we investigate here is whether the arterial vs. venous origin of the ECs might regulate mural cell recruitment and vessel assembly. Thus, we utilized human aortic ECs, due to their arterial origin, to assess whether they may be capable of recruiting CASMCs, while pericyte recruitment might be reduced, since the human aorta contains many concentric layers of VSMCs and few if any pericytes. Co-cultures were established with aortic ECs and either CASMCs or pericytes, and after 72 h, the cultures were fixed to quantify mural cell recruitment. Interestingly, aortic ECs formed networks of tubes, like the HUVECs and, also strongly recruited pericytes, while CASMCs showed much less responsiveness and recruitment (Figures 6A, D). The aortic EC tubes were narrower and more elongated with increased lengths in the presence of pericytes compared to CASMCs (Figures 6B, C). We also pre-treated the aortic ECs with VEGF to prime them, and under these conditions, the same results were observed with strong pericyte recruitment to the tube networks, while again, the CASMCs showed minimal recruitment (Figures 6A, D).

**FIGURE 7:**
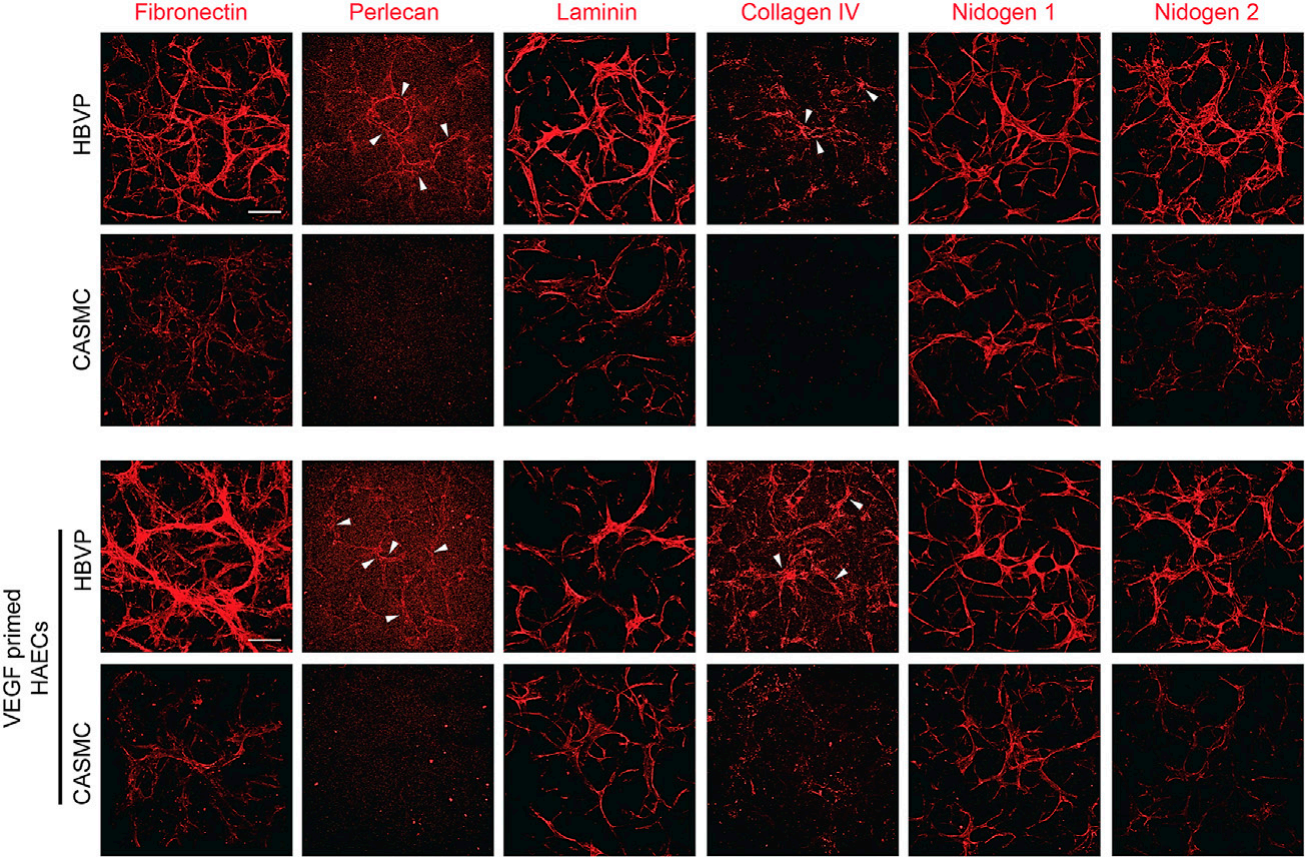
Pericyte recruitment to developing aortic EC tube networks markedly induces basement membrane matrix protein deposition, while coronary artery smooth muscle cells fail to recruit leading to diminished basement membrane deposition. Human aortic endothelial cells (HAECs) were utilized for these experiments and were either left untreated or primed with vascular endothelial cell factor (VEGF). Untreated aortic ECs or VEGF-primed aortic ECs primed were co-cultured with either GFP-labeled pericytes or coronary artery smooth muscle cells (CASMCs) in 3D collagen matrices and then fixed at 120 h. Immunostaining for the indicated basement membrane matrix proteins was performed in the absence of detergent and the stained cultures were imaged by confocal microscopy to assess extracellular deposition of basement membrane components. Arrowheads indicate deposition of perlecan or collagen type IV. Bar equals 200 μm.

**FIGURE 8:**
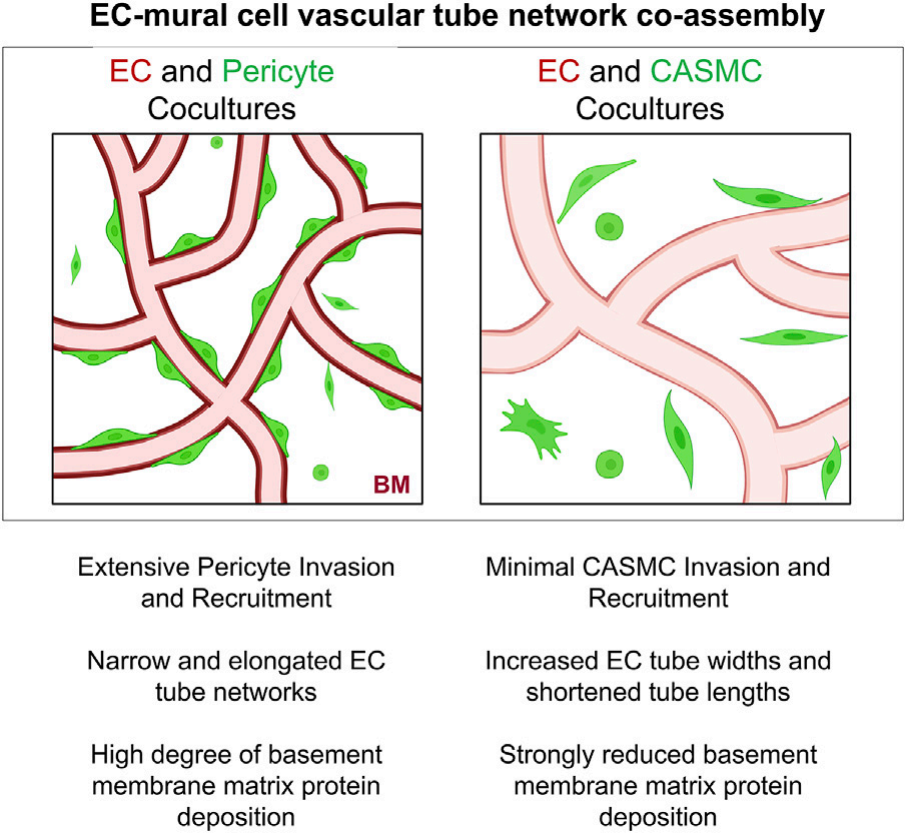
Schematic illustrating differences in the ability of pericytes to invade 3D matrices, and recruit to, remodel, and induce basement membrane matrix assembly around developing EC-lined tubes compared to vascular smooth muscle cells. Key findings are summarized demonstrating functional differences between pericytes versus coronary artery smooth muscle cells in their abilities to co-assemble and remodel developing EC-lined tube networks, a required step controlling vascular development. BM indicates basement membrane.

Finally, we evaluated vascular basement membrane deposition in the aortic EC-pericyte co-cultures compared to the aortic EC-CASMC co-cultures (Figure 7). Immunostaining of the basement membrane matrix components, fibronectin, perlecan, laminin, collagen type IV, nidogen 1, and nidogen 2, revealed much greater deposition in the EC-pericyte co-cultures compared to the EC-CASMC co-cultures (Figure 7). Overall, our findings show that pericytes strongly respond to ECs undergoing tubular morphogenesis whether they are derived from either venous or arterial sources. In either case, this occurs when the different ECs are untreated or primed with VEGF, whereby the pericytes recruit to tubes and induce vascular basement membrane deposition (Figure 8). In contrast, CASMCs show minimal responsiveness to these different types of ECs forming tubes and, furthermore, they demonstrate poor recruitment with minimal basement membrane matrix deposition compared to pericytes (Figure 8). Thus, our data reveals that differentiated VSMCs, such as CASMCs, do not appear to be capable of this type of pericyte behavior. Overall, this work suggests that pericytes or pericyte-like precursors of VSMCs will be required for assembly of arteries and veins in the blood vascular system.

## Discussion

In this study, we investigate key functional differences between pericytes and vascular smooth muscle cells (VSMCs) during EC-mural cell tube assembly under defined culture conditions in 3D collagen matrices. Blood vessels require mural cell support and the similarities or distinctions between how these different mural cells are recruited to EC-lined tubes is not completely understood. Using our defined culture and growth factor conditions, we have previously described the ability of pericytes (human or bovine) to recruit and assemble along the basal surface of forming EC tube networks (Stratman et al., 2009; Stratman et al., 2010; Stratman and Davis, 2012; Kemp et al., 2020). Furthermore, we have reported that ECs derived from human umbilical vein, dermal microvascular, umbilical artery, and iPS-derived sources form tubes in 3D matrices, and also recruit pericytes under these conditions (Stratman et al., 2009; Stratman et al., 2011; Bowers et al., 2016). Pericyte recruitment to these tubes in each case results in capillary basement membrane matrix deposition. EC tubes alone or interference with pericyte recruitment during co-culture assays leads to markedly diminished basement membrane deposition with widened tube widths and shortened tube lengths (Stratman et al., 2010; Bowers et al., 2020; Kemp et al., 2020).

In this report, we have directly compared the ability of human pericytes vs. human CASMCs to respond to known EC-derived factors that affect pericyte recruitment and, also to co-assemble with different types of EC tube networks. Remarkably, we find that pericytes selectively recruit to assembling EC tube networks compared to CASMCs, which show little ability to perform this function. For these studies, we have utilized HUVECs, which are of venous EC origin, but we have also performed this work by pre-treating these ECs with VEGF (i.e., VEGF priming), a known stimulus for arteriogenesis (Lawson et al., 2002). In addition, we utilize human aortic ECs (of arterial origin) and, also prime them or not with VEGF to perform EC-mural cell co-culture assays. In each instance, pericytes selectively recruit to each of these different types of EC tube networks and this recruitment regulates both vessel remodeling and basement membrane deposition. Pericyte recruitment to EC tubes induces basement membrane assembly and this also results in vessel remodeling responses including more elongated and narrow tube networks. In contrast, CASMCs fail to recruit or regulate vessel remodeling and basement membrane deposition in EC-CASMC co-cultures. This lack of CASMC recruitment led to less elongated tubes and wider tube widths and much reduced basement membrane matrix deposition. Overall, our functional data comparing these different mural cell types reveals the surprising result that CASMCs show markedly less ability to recruit to EC-lined tubes, compared to pericytes, and they also demonstrate markedly reduced invasive ability in response to defined factors including the PDGF isoforms, PDGF-BB and PDGF-DD, as well as ET-1. These functional data suggest the strong possibility that differentiated CASMCs and VSMCs in general, are not likely to be the mural cell type that recruits to different types of EC tubes (i.e., arteries and veins) during developmental vessel assembly. Our results suggest that pericytes may be the mural cell type that performs this essential EC-mural cell tube co-assembly function.

An important possibility is that pericytes recruit to different tube networks and then in the case of arteries and veins, receive other stimuli, such as flow forces (Majesky, 2018), growth factors [e.g., TGFβ (Hirschi et al., 1998)] or other differentiating agents, such as retinoic acid (Miano and Berk, 2000), which leads to further maturation of pericytes into the VSMC lineage. Genetic analyses using RNA sequencing approaches have led to the conclusion that the two mural cell lineages are related. Our qPCR genetic analyses comparing pericytes to CASMCs reveals similarities, but also differences such as higher mRNA expression of *cspg4*, *pdgfrb*, and *ednra* in pericytes. The latter two receptors control pericyte invasion responses to PDGF isoforms and ET-1, which are the key EC-derived factors affecting this response as well as recruitment to EC tubes (Kemp et al., 2020). CASMCs show much higher mRNA expression of *eln*, *tbx3*, *lox*, *adamts4, tgm2*, and *prg4* compared to pericytes. Interestingly, *notch3* was more highly expressed by pericytes as well as known smooth muscle cell target genes including *tagln*, *cald1*, *cnn1*, *smtn*, and *acta2*. These latter genes further, suggest a possible cell lineage relationship between pericytes and CASMCs. Interestingly, both cell types express comparable levels of *timp3*, a matrix metalloproteinase (MMP), and a disintegrin and metalloproteinase (ADAM) proteinase inhibitor, which has a restricted expression profile, again suggesting a relationship between the two lineages.

An important study using lineage tracing approaches revealed that pericytes appear to be a developmental precursor of VSMCs (Volz et al., 2015). This conclusion is strongly supported by the functional studies in this manuscript, whereby pericytes show a selective ability to recruit to developing tubes compared to CASMCs. This lineage tracing study was performed using CSPG4 and PDGFRβ promoters (Volz et al., 2015), which are more highly expressed in pericytes relative to CASMCs in our study, and which are known to show greater expressivity in pericytes. In both the developing coronary and kidney vasculature, it was demonstrated that VSMCs develop from a pericyte precursor that can be labeled by activating either the CSPG4 or PDGFRβ promoters early enough to label the pericytes prior to the appearance of VSMCs in these tissue beds (Volz et al., 2015). In each case, the VSMCs were derived from a pericyte precursor. They also demonstrated that Notch3 knockout leads to blockade in the transition of pericytes to VSMCs in mice but did not affect pericyte development or the recruitment of pericytes to developing tubes *in vivo* (Volz et al., 2015). Other investigators have confirmed this conclusion that Notch3 is important, along with Notch2, for VSMC development using similar knockout approaches (Ando et al., 2019). In addition, the developing mural cells with the coronary vasculature are known to arise from a common precursor from the epicardium, and, thus, both pericytes and VSMCs appear to be derived from these precursors (Riley and Smart, 2011; Wu et al., 2013; Volz et al., 2015; Quijada et al., 2020). In our view, this provides additional support for the concept that pericytes may represent the developmental precursor of VSMCs.

The functional studies presented in this work provide further support for this concept. The implication of our work suggests that pericytes recruit to developing EC tube networks, and remain pericytes in the context of capillary networks, but would then further differentiate into VSMCs in arteries and veins in response to unique stimuli within these distinct vascular beds. Overall, our new data provides considerable evidence that the two mural cells, pericytes and CASMCs, despite genetic similarities, are functionally distinct in their abilities to recruit to EC-lined tubes, affect EC tube remodeling, and induce deposition of basement membrane matrices (Figure 8). These critical functions, which are necessary for vascular development, appear to be a selective ability of pericytes; a cell type that is known to regulate vascular morphogenesis, vascular remodeling, vascular stabilization, and vascular regeneration following a variety of tissue injuries (Armulik et al., 2011; Berthiaume et al., 2018; Kemp et al., 2022).

## Data Availability

The original contributions presented in the study are included in the article/Supplementary Material, further inquiries can be directed to the corresponding author.
